# Affinity profiling of monoclonal antibody and antibody-drug-conjugate preparations by coupled liquid chromatography-surface plasmon resonance biosensing

**DOI:** 10.1007/s00216-018-1414-y

**Published:** 2018-10-17

**Authors:** Dina Lakayan, Rob Haselberg, Rabah Gahoual, Govert W. Somsen, Jeroen Kool

**Affiliations:** 1 Division of Bioanalytical Chemistry, Amsterdam Institute for Molecules, Medicines and Systems, Department of Chemistry and Pharmaceutical Sciences, Faculty of Science, Vrije Universiteit, De Boelelaan 1085, 1081 HV Amsterdam, The Netherlands; 2 TI-COAST, Science Park 904, 1098 XH Amsterdam, The Netherlands; 3 Unité de Technologies Chimiques et Biologiques pour la Santé, Faculté de Pharmacie, Université Paris Descartes, 4 avenue de l’observatoire, 75270 Paris Cedex 06, France

**Keywords:** Surface plasmon resonance, Online coupling, Size exclusion chromatography, Cation exchange chromatography, Trastuzumab, Biopharmaceutical antibody-drug conjugates

## Abstract

Monoclonal antibodies (mAbs) and antibody-drug conjugates (ADCs) are highly potent biopharmaceuticals designed for targeted cancer therapies. mAbs and ADCs can undergo modifications during production and storage which may affect binding to target receptors, potentially altering drug efficacy. In this work, liquid chromatography was coupled online to surface plasmon resonance (LC-SPR) to allow label-free affinity evaluation of mAb and ADC sample constituents (size and charge variants), under near-native conditions. Trastuzumab and its ADC trastuzumab emtansine (T-DM1) were used as a test sample and were analyzed by aqueous size-exclusion chromatography (SEC)-SPR before and after exposure to aggregate-inducing conditions. SEC-SPR allowed separation of the formed aggregates and measurement of their affinity towards the ligand-binding domain of the human epidermal growth factor receptor 2 (HER2) receptor immobilized on the surface of the SPR sensor chip. The monomer and aggregates of the mAb and ADC were shown to have similar antigen affinity. Conjugation of drugs to trastuzumab appeared to accelerate the aggregate formation. In addition, cation-exchange chromatography (CEX) was coupled to SPR enabling monitoring the maximum ligand-analyte binding capacity (*R*_max_) of individual charge variants present in mAbs. Deamidated species and lysine variants in trastuzumab sample were separated but did not show different binding affinities to the immobilized HER2-binding domain. In order to allow protein variant assignment, parallel MS detection was added to the LC-SPR setup using a column effluent split. The feasibility of the LC-MS/SPR system was demonstrated by analysis of trastuzumab and T-DM1 providing information on antibody glycoforms and/or determination of the drug-to-antibody ratio (DAR), while simultaneously monitoring binding of eluting species to HER2.

**Electronic supplementary material:**

The online version of this article (10.1007/s00216-018-1414-y) contains supplementary material, which is available to authorized users.

**Graphical abstract:**
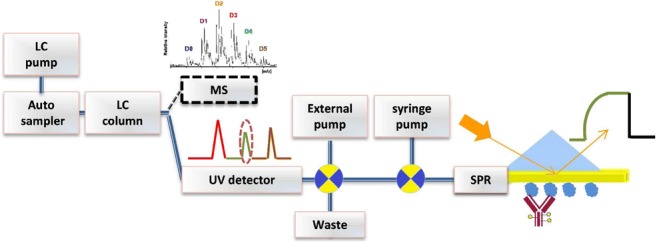
ᅟ

## Introduction

Over the last two decades, monoclonal antibodies (mAbs) have found vast application as therapeutic agents for cancer treatment [1–4]. The specific binding of mAbs to tumor cell-surface antigens (receptors) leads to less endogenous ligand-receptor interactions and subsequently inhibition of the downstream signaling [2]. The highly selective binding properties of mAbs have also made them a valuable tool for drug delivery [5, 6]. Development of antibody-drug conjugates (ADCs) was initiated by conjugation of chemotherapeutic drugs to murine antibodies, and has been later improved by producing conjugates of humanized mAbs and radionuclides or cytotoxic drugs, resulting in potent targeted therapeutic agents [3].

mAbs and ADCs can undergo physical and chemical changes through the manufacturing, formulation, and storage processes, resulting in aggregation and degradation products in these samples. The presence of these products in pharmaceutical samples is continuously studied in the biopharmaceutical industry using different analytical, biochemical, and biological methods, allowing assessment of biological and/or biochemical properties of complete preparations. Recently, high-performance and multidimensional protein separation techniques have been introduced as powerful tools for the compositional characterization of mAbs and ADCs [7–10]. Nonetheless, monitoring the biological activity of individual components, such as aggregates and degradation products, in these products is a challenge due to their low concentration and their potential reversible behavior.

The affinity properties of mAbs and ADCs have been studied using surface plasmon resonance (SPR) [11, 12]. However, stand-alone SPR cannot distinguish the affinity of individual sample components [13] such as size and charge variants of mAb and ADC samples. Offline affinity monitoring of collected fractions after chromatographic separation (e.g., liquid chromatography (LC)), however, is time-consuming and may be difficult when concentrations are low or aggregation states are affected by the fractionation. Hence, the direct online coupling of LC to SPR could be an attractive approach for affinity studies of protein sample constituents [14]. However, SPR ligand-analyte affinity monitoring is only possible when eluting analytes are preserved in their native (non-denatured) form. For this reason, preservation of analytes in their native state during separation is a crucial factor when selecting a separation technique prior to SPR. Native separation of size, charge, and hydrophobic variants in principle can be performed by, e.g., size-exclusion chromatography (SEC) [15–18], ion-exchange chromatography (IEC) [19, 20], and hydrophobic interaction chromatography (HIC), respectively.

In this study, an LC-SPR approach was investigated for online affinity evaluation of aggregates and charge variants present in mAbs and ADCs. The antibody trastuzumab and its ADC, trastuzumab emtansine (T-DM1), were used as a test sample. These two compounds target human epidermal growth factor receptor 2 (HER2), which is a receptor overexpressed in 25 to 30% of breast cancers [21]. Trastuzumab blocks the HER2 dimerization and eventually stops the intracellular signaling and cell proliferation [22]. T-DM1 is an ADC in which the cytotoxic agent DM1 is coupled covalently through thioether bonds to trastuzumab [23–25]. Binding of T-DM1 to HER2 is followed by internalization of the T-DM1 and release of cytotoxic agents within cancer cells, eventually leading to cell apoptosis [26, 27]. The affinity and binding kinetics of trastuzumab and T-DM1 to HER2 were first studied using stand-alone SPR. Next, an SEC-SPR method was established allowing online monitoring of the affinity of the same mAb and ADC samples. The SEC-SPR method was applied to stressed mAb and ADC samples probing the affinity of formed aggregates and compare it to simultaneously acquired monomer affinity. In addition, an online cation exchange chromatography (CEX)-SPR method was developed and evaluated by studying charge variants present in mAbs and their maximum binding capacity of towards the immobilized ligand on the surface of SPR sensor. Finally, SEC-SPR with parallel MS detection was established and its potential was investigated by the analysis of trastuzumab and T-DM1 samples.

## Materials and methods

### Chemicals and reagents

Herceptin (trastuzumab) was from Roche (Basel, Switzerland) and Kadcyla (ado-trastuzumab emtansine (T-DM1)) from Genentech Inc. (South San Francisco, CA, USA). Human HER2/ErbB2s protein (His tagged) was obtained from Sino Biologicals (North Wales, USA). Ethylenediaminetetraacetic acid (EDTA), 2-[4-(2-hydroxyethyl)piperazin-1-yl]ethanesulfonic acid (HEPES), Tween 20, disodium hydrogen phosphate dihydrate, phosphate-buffered saline (PBS) tablets, ethanolamine hydrochloride, *N*-hydroxy succinimide (NHS), *N*-(3-dimethylaminopropyl)-*N*′-ethylcarbodiimide hydrochloride (EDC), hydrochloric acid 37% (HCl), acetic acid (≥ 99%), glycine-HCl, ammonium acetate, sodium hydroxide (NaOH), sodium chloride (NaCl), and sodium acetate were purchased from Sigma-Aldrich (Steinheim, Germany). Sodium azide (toxic, should be discarded in hazardous waste) was obtained from Mallinckrodt Baker B.V. (Deventer, the Netherlands). β-mercaptoethanol was obtained from Merck (Darmstadt, Germany). Deionized water was produced by a Milli-Q purification system from Millipore (Amsterdam, the Netherlands).

### Sample preparation

Trastuzumab and T-DM1 formulations were diluted in SPR running buffer or LC mobile phase to the desired concentrations. Stress samples were prepared by incubating 100 μL of 20 mg/mL trastuzumab and T-DM1 formulations at 60 °C in an Eppendorf ThermoMixer (Hauppauge, NY, USA) at 300 rpm for 3 days. The sample volumes for the stressed samples were then corrected by adding SEC mobile phase up to the volume of 100 μL followed by incubation for 14 h. Next, samples were diluted to the desired concentration for analysis.

### Surface plasmon resonance conditions

SPR analyses and detection were performed with a multi-parametric SPR Navi 210 instrument from BioNavis Ltd. (Tampere, Finland) employing a 670-nm laser in angular scan mode. The flow cell temperature was 20 °C for all the experiments. The liquid flow was 30 μL/min for the conventional SPR measurements (except for the immobilization step). The immobilization buffer (pH 7.4) used for the sensor chip preparation (see below) contained 10 mM HEPES, 150 mM NaCl, 3 mM EDTA, and 0.005% Tween 20 (*v*/*v*). PBS tablets were used for preparing the running buffer (pH 7.4) for the stand-alone SPR analysis (see below). The tablets contain 0.01 M phosphate buffer with 2.7 mM potassium chloride and 0.137 M sodium chloride. Ten millimolar of glycin-HCl (pH 1.5) was used as regeneration solution. Sample injections were performed with full-loop (250 μL) injection setup, with air plug injections before and after sample collection to avoid sample dilution.

For HER2 sensor preparation, carboxymethyl dextran (CMD) hydrogel gold sensor chips (BioNavis) were equilibrated with immobilization buffer for approximately 10 min. After getting a stable baseline, the surface was activated with a solution containing 0.4 M EDC and 0.1 M NHS for 7 min in the sample channel and the reference channel. This was followed by a 10-min injection of 20 μg/mL HER2 in 10 mM sodium acetate (pH 4) at a flow rate of 10 μL/min in the sample channel, while immobilization buffer was directed to the reference channel. A typical sensorgram obtained during immobilization is shown in Fig. S1 in the Electronic Supplementary Material (ESM). As result of the immobilization of the receptor on the SPR sensor, the refractive index of the surface changes which caused a shift of the SPR angle. A total shift of 0.64 degrees is observed after the immobilization (ESM Fig. S1). After the immobilization step, both channels were subsequently deactivated with 1 M ethanolamine hydrochloride (pH 8) two times for 7 min with a 1-min buffer flow in between the injections. As a result, all the remaining active esters were subsequently deactivated. The surface was then regenerated with regeneration solution two times for 1 min with a 1-min buffer flow to the surface in between the two regeneration steps. Correspondingly, all non-covalently bound receptor was removed by two consecutive regeneration steps, resulting in a remaining total response of 0.45 degrees. This corresponds to 270 ng/cm^2^ immobilized receptor on the surface, assuming an angle shift (at 670 nm) is corresponding to about 6000 resonance units (RU) and 1000 RU ≈ 1 ng/mm^2^ [28].

For stand-alone SPR analysis, HER2 was first immobilized on the SPR sensor chip. Subsequently, the prepared sensor chip (with immobilized HER2) was kept in a bottle containing running buffer while the SPR instrument was equilibrated with the same running buffer. After this, the prepared sensor chip was inserted into the instrument. After obtaining a stable baseline, the interaction between immobilized HER2 and 0.1–100 μg/mL of trastuzumab and T-DM1 antibody diluted in the running buffer was studied. Samples were plug injected for 4 min. The surface was regenerated with the regeneration solution in between analyses (injected for 1 min).

### LC conditions

All LC analyses were carried out using a Shimadzu LC system (‘s Hertogenbosch, the Netherlands) comprising an LC-20AB binary system pump, a UV detector SPD-20A, and an autosampler SIL-20AC. Elution was monitored using UV absorption at 280 nm.

SEC was performed using a TSKgel G3000SWxl (7.8 × 300 mm) column from TOSOH Bioscience (Griesheim, Germany). Separations were done in an isocratic mode using 0.01 M phosphate buffer (pH 7.4) containing 2.7 mM potassium chloride and 0.137 M sodium chloride as mobile phase at a flow rate of 0.3 mL/min. Native and stressed mAb and ADC samples were prepared in the mobile phase and kept in the cooled autosampler at 10 °C before injection for 2 h prior to the injection. The injection volume was 12 μL throughout all SEC experiments.

For CEX analyses, a Bakerbond Wide-Pore Carboxy-Sulfon (Csx) 5xM (4.6 × 250 mm) cation exchange column from J.T. Baker (Deventer, the Netherlands) was used. Separations were done using gradient mode. The mobile phase flow rate was 0.5 mL/min and the column temperature was kept at 40 °C. Mobile phase A was 200 mM disodium hydrogen phosphate (pH 6.9, adjusted with HCl), while mobile phase B was 200 mM disodium hydrogen phosphate (pH 6.9) with 2 M NaCl. The linear gradient from 10 to 45% of mobile phase B was applied from 0 to 55 min. From 55 to 60 min, the mobile phase composition was changed to 100% mobile phase B and, after that, it changed to the starting conditions (10% B) in 60 min at which it remained for 30 min until the end of the run. Trastuzumab samples were prepared in the mobile phase and kept in autosampler (4 °C). Fifty microliters of each sample was injected.

### On-line LC-SPR

**Fig. 1:**
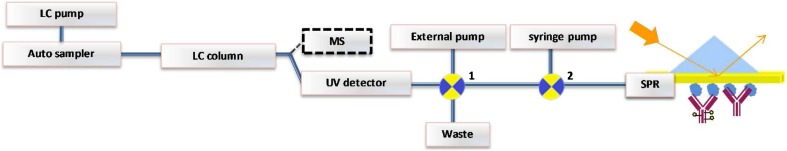
Schematic representation of the online LC-SPR system. LC separation of biomacromolecules by, e.g., SEC or CEX is followed by successive online UV absorbance and SPR detection. An external switch valve 1 and pump is used for optional heart-cutting analysis. The SPR sensor is regenerated in between the affinity analysis, employing external switch valve 2 and syringe pump. Optional parallel MS detection can be performed using a post-column split

The LC-SPR setup is schematically presented in Fig. 1. Low dead volume tubing (250 μm ID blue PEEK tubing; IDEX Health and Science) was used to connect the LC and the SPR as well as the switch valves. The outlet tubing from the UV detector was connected to an external switch valve 1 (Spark Holland, Emmen, the Netherlands), which was automated to switch in between injections while an external pump (Knauer K-500, Berlin, Germany) kept a constant flow of mobile phase (same flow rate as from LC pump) when performing heart-cutting of an eluting peak or fraction of interest. To avoid possible carryover during the column equilibration, switch valve 1 was used for heart-cutting the native sample’s main peak, in between injections. The switch valve was controlled by in-house developed software (Ariadne). The outlet of the switch valve 1 was connected to switch valve 2 in the BioNavis MP-SPR instrument autosampler and then directed to the flow cell. The flow was led from the sample channel to the reference channel in a serial mode using the dedicated injection setup of the BioNavis MP-SPR instrument. Switch valve 2 was switched in between each analysis for regeneration of the sensor chip surface, using the SPR Navi autosampler software. Sixty microliters of the regeneration solution was injected through the existing loop in BioNavis autosampler and was directed with the LC flow to the sensor surface for 10 s in between injections. The eluted peaks were monitored with the UV detector in chromatograms. The resonance angle shift was monitored in time and plotted in a sensorgram.

Using the SEC-SPR setup, the interaction of eluting native trastuzumab and T-DM1 (1–1500 μg/mL) with the immobilized HER2 on the SPR sensor chip was monitored. For stressed trastuzumab samples, monomer and aggregate peaks were SPR-analyzed for injected total sample concentrations of 10–60 μg/mL and 500–3000 μg/mL, respectively. For stressed T-DM1 samples, monomer and aggregate peaks were analyzed for injected total sample concentrations of 50–300 μg/mL and 500–3000 μg/mL, respectively. The surface was regenerated in between injections, as described above.

Using the CEX-SPR setup, the interaction of charge variants’ presence in trastuzumab samples (1–200 μg/mL) with the immobilized HER2 on the sensor chip was monitored by SPR. Selected peaks were heart-cutted to SPR using switch valve 1 in order to determine the *R*_max_ for individual charge variants. In order to obtain affinity curves by heart-cutting analysis, trastuzumab concentrations of 10–2000 μg/mL were injected. For all analyses, the gradient flow from 50 to 90 min was directed to the waste. Switch valve 2 was used for regeneration of the surface in between injections, as described above.

### SEC-SPR/MS

The LC-SPR/MS setup (which employed here for SEC) is schematically presented in Fig. 1. An Ultimate3000 Rapid Separations UHPLC system (Thermo Scientific/Dionex, Breda, the Netherlands) equipped with quaternary high-pressure gradient solvent delivery pumps, a temperature controlled autosampler, and a column oven was used. The eluent was composed of 75 mM ammonium acetate (pH 6.9). For trastuzumab, a Waters ACQUITY UPLC® BEH200 SEC column (4.6 × 300 mm; 1.7-μm particle size) was used with a flow rate of 0.3 mL/min. For T-DM1, a Tosoh TSKgel G3000SWxl column (7.8 × 300 mm; 5-μm particle size) was used with a flow rate of 0.25 mL/min. UV absorbance was at 280 nm using a 759A Absorbance UV-Vis detector from Applied Biosystems (Foster City, CA, USA). For SEC-SPR/MS analysis, 10 μL of trastuzumab or T-DM1 (20 mg/mL) was injected. The SEC column effluent was split 1:50, and the minor flow was directed to the ESI source of the mass spectrometer. The major flow was led via the UV detector followed by the SPR instrument.

MS detection was performed with a Maxis HD mass spectrometer (Bruker Daltonics, Bremen, Germany). MS parameters were optimized for the ions of interest to allow their efficiently transfer to the time-of-flight (TOF) analyzer without causing fragmentation. For MS optimization, trastuzumab (0.75 mg/mL) in 50 mM ammonium acetate (pH 6.9) was infused to the MS at a flow rate of 4 μL/min. Final ESI source parameters were as follows: ESI voltage, − 4.3 kV; nebulizing gas pressure, 0.6 bar; dry gas was 4.2 L/min and source temperature was 140 °C; ion funnels were set at 400 Vpp. Mass spectra were recorded in the *m*/*z* 2000–9000 range using a sampling rate of 0.5 Hz. DataAnalysis software version 4.2 (Bruker Daltonics) was used. MS spectra were deconvoluted using maximum entropy algorithm [29] which was part of the data analysis software.

### SPR data analysis

Resonance angle shifts were monitored over time and plotted in sensorgrams. Total SPR binding was plotted by subtracting the sample channel-binding rate from the reference channel to correct for non-specific surface binding or bulk effects. The evaluation of kinetic constants was performed with the TraceDrawer software (Version 1.7, Ridgeview Instruments AB, and Sweden) using a bivalent interaction model fit. Affinity curves were plotted using GraphPad PRISM Software (San Diego, CA, USA).

## Results and discussion

An experimental LC-SPR setup (Fig. 1) was used allowing separation of sample components (based on, e.g., their size or charge) prior to measuring their affinity towards an immobilized antigen on a sensor surface monitored by SPR. An optional effluent split provided the possibility to perform parallel MS detection for protein characterization. The performance of the developed LC-SPR methodology was evaluated by the analysis of the therapeutic antibodies, trastuzumab, and T-DM1.

### Stand-alone SPR analysis

Binding of native trastuzumab and T-DM1 to the immobilized HER2 on the SPR sensor chip was studied by triplicate plug injection of different concentrations and monitoring the shift in resonance-dip angle over time, creating a sensorgram. For both samples, the SPR signal remained elevated in time after injection, confirming high-affinity binding. After each sample injection, regeneration solution was injected for 1 min resulting in the complete return of the signal to baseline. From the obtained affinity curves (ESM Fig. S2), the association (*k*_a_) and dissociation (*k*_d_) rate constants and the dissociation constant (*K*_D_ = *k*_d_/*k*_a_) were calculated for the analyzed antibodies (ESM Table S1). The results showed quite similar *k*_a_ and *k*_d_ values for trastuzumab and T-DM1, with *K*_D_ values of 1.8 ± 0.15 nM and 2.7 ± 0.14 nM, respectively, which is in line with previous reports [30].

### SEC-SPR of trastuzumab and T-DM1

To evaluate the affinity of potential size variants of with HER2, trastuzumab, T-DM1, and their stressed samples were analyzed by SEC-SPR. An aqueous mobile phase, similar to the stand-alone SPR running buffer, was used to keep the analytes and the immobilized ligand on the SPR sensor chip surface as close as possible to their native state.

**Fig. 2:**
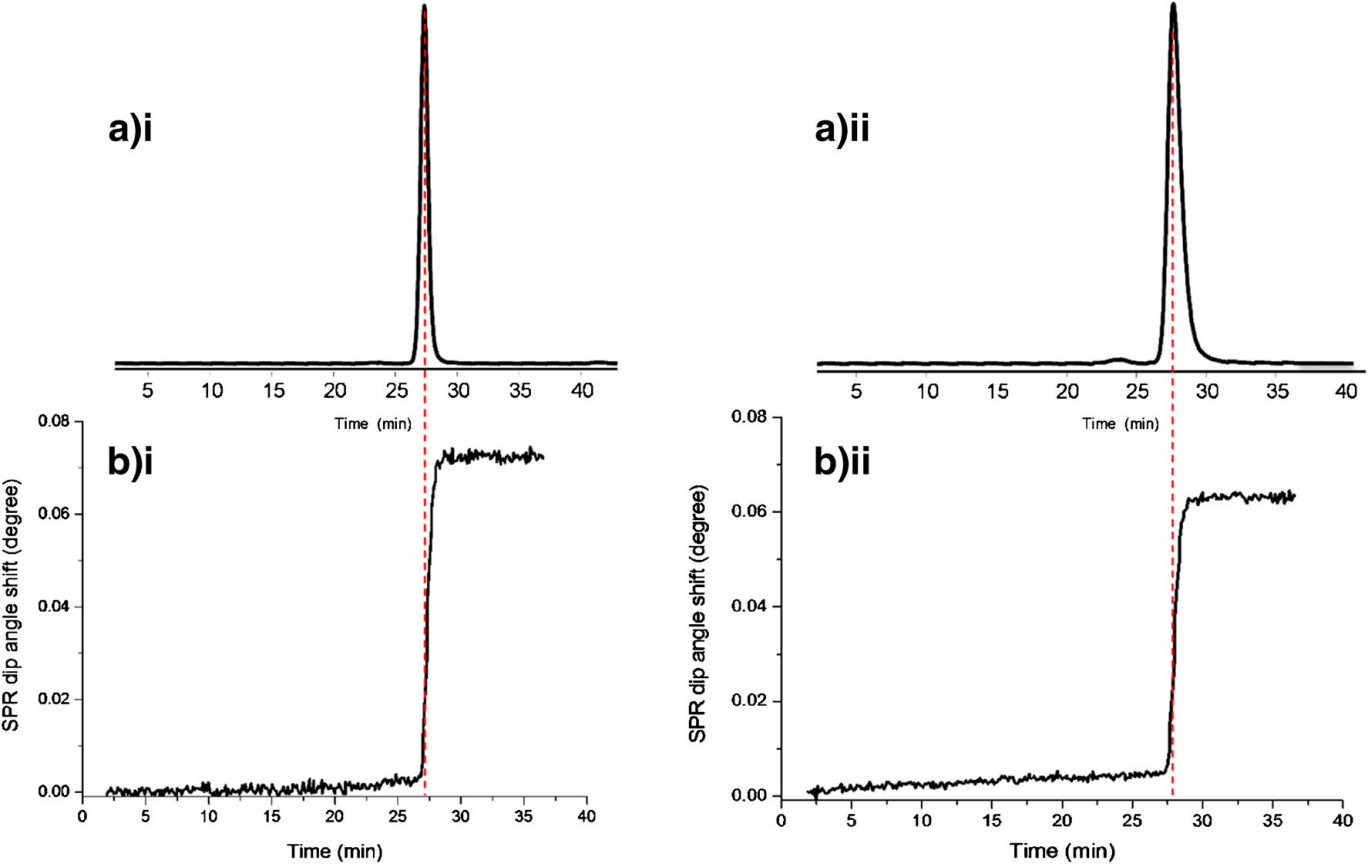
SEC-UV-SPR of (1 mg/mL) trastuzumab (i) and T-DM1 (ii). (a) UV chromatogram. (b) SPR sensorgram

During the SEC-UV analysis (Fig. 2(a.i, b.i)) of trastuzumab, one antibody peak was observed. For T-DM1, a small band (retention time, 23 min) prior to the main peak was observed, indicating the presence of high-molecular-weight species (HMWs) in this sample. The refractive index changes due to the interaction of eluted analytes on the SPR surface were then monitored in time. For the same samples, SEC-SPR was performed by directing the LC effluent to the SPR flow cell using switch valve 1 (Fig. 1). For both samples (Fig. 2(a.ii, b.ii)), a clear increase of the SPR signal was observed at the retention times of the respective antibodies. The SPR signal remained elevated after complete elution confirming the high affinity of the eluted antibodies to HER2 on the surface of the sensor chip. The lower maximum SPR signal as compared to stand-alone analysis can be explained by the significant analyte dilution caused by the SEC process, as also described previously [14]. Next to the main peak, no other binding components were observed with SPR detection for both samples. After complete elution of the antibody proteins, the column effluent flow was switched to waste and mobile phase was pumped to the SPR cell using switch valve 1, thereby avoiding exposure of the sensor to lower-molecular-weight sample components. The SPR sensor surface was regenerated after each analysis using switch valve 2. The regeneration time (10 s) was optimized by monitoring the SPR signal return to baseline. Each sensor chip could be normally used for about 100 analyses with regeneration steps in between, showing a signal decrease of less than 20% over time.

The calibration (UV absorbance peak area vs. concentration) and affinity curves (SPR response after complete elution vs. concentration) were obtained by injection of trastuzumab and T-DM1 at different concentration (ESM Fig. S3). The *k*_a_, *k*_d_, and *K*_D_ values were estimated from the obtained curves regardless of the mass transfer limitation and diffusion coefficient (ESM Table S1). Trastuzumab and T-DM1 showed similar *K*_D_ values of 0.16 ± 0.05 nM and 0.19 ± 0.06 nM, respectively; also, stand-alone analysis indicated no significant difference. The approximately 10 times lower absolute *K*_D_ values as obtained with stand-alone analysis can be explained by the chromatographic dilution, which results in lower actual analyte concentration at the sensor surface. Additionally, the higher flow rate used in SEC-SPR analysis will reduce the binding time of the analyte to the antigen [14].

**Fig. 3:**
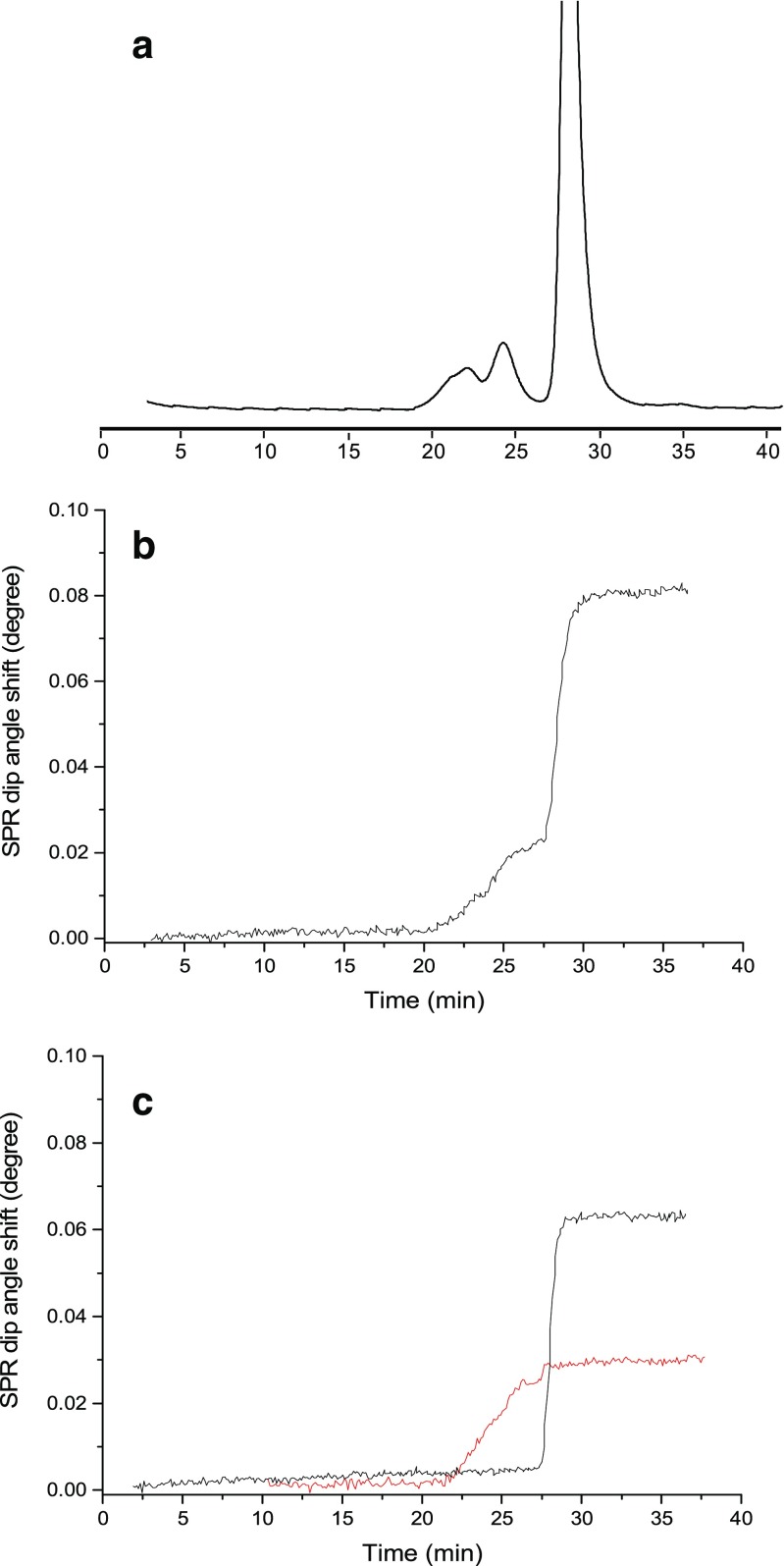
SEC-UV-SPR of stressed T-DM1 sample (1 mg/mL). **a** UV chromatogram, **b** SPR sensorgram, and **c** SPR sensorgrams obtained after heart-cutting of the HMW bands (19–26 min) (red) and the main peak (28–29 min) (black)

Subsequently, antibody samples stressed at 60 °C for 14 h were analyzed by SEC-UV-SPR. The resulting UV chromatograms showed peaks eluting before the main protein peak, indicating the presence of HMW species (aggregates) in both the stressed trastuzumab and T-DM1 sample (Fig. 3a). Under these conditions, the relative peak area of the aggregates in the stressed trastuzumab and T-DM1 samples was approximately 3% and 10%, respectively. The relatively higher concentration of aggregates for the heat-stressed T-DM1 sample was expected due to the drug conjugation to the antibody cysteines, resulting in a less-stable CH2 domain [31]. The SEC-SPR sensorgram of the stressed T-DM1 sample (Fig. 3b) indicates that the present aggregate species bind to the immobilized HER2. The same was observed for the aggregates in the stressed trastuzumab sample. The binding of aggregate and monomer could be studied independently by heart-cutting the separate peaks employing switch valve 1 (Fig. 3c) and directing them to the SPR flow cell with a clean sensor surface. Clearly, the antibody aggregates bind significantly to the immobilized ligand on the sensor surface.

**Fig. 4:**
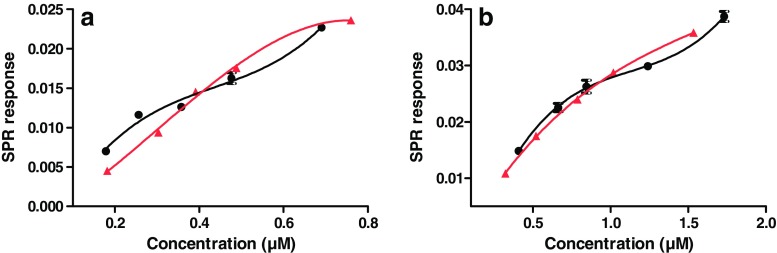
SPR affinity curves of monomer (red) and aggregate (black) species observed during LC-UV-SPR of samples of stressed **a** trastuzumab and **b** T-DM1. For stressed trastuzumab samples, total sample concentrations of 10–60 μg/mL and 500–3000 μg/mL were injected for analysis of monomer and aggregate peaks, respectively. For stressed T-DM1 samples, total sample concentrations of 50–300 μg/mL and 500–3000 μg/mL were injected for analysis of monomer and aggregate peaks, respectively

In order to assess the relative affinity of the aggregates with respect to the monomer, stressed antibody samples with relatively high concentrations of aggregates were prepared and analyzed by SEC-UV-SPR. The monomer and aggregate concentrations were determined from the UV peak areas using calibration curves obtained from the native antibodies. The maximum SPR signal obtained for the heart-cutted peaks was plotted versus the concentration (Fig. 4). The resulting affinity curves show very similar affinities for the monomer and the aggregates for both trastuzumab and T-DM1. The aggregate curves showed a quasi sigmoidal pattern upon fitting model, indicating the potential presence of species of different affinity in the sample [32]. The here-presented data showed the suitability of the SEC-SPR analysis developed for affinity monitoring of size variants towards their appropriate ligand immobilized on the SPR sensor, in the therapeutic samples.

### CEX-SPR of Trastuzumab

**Fig. 5:**
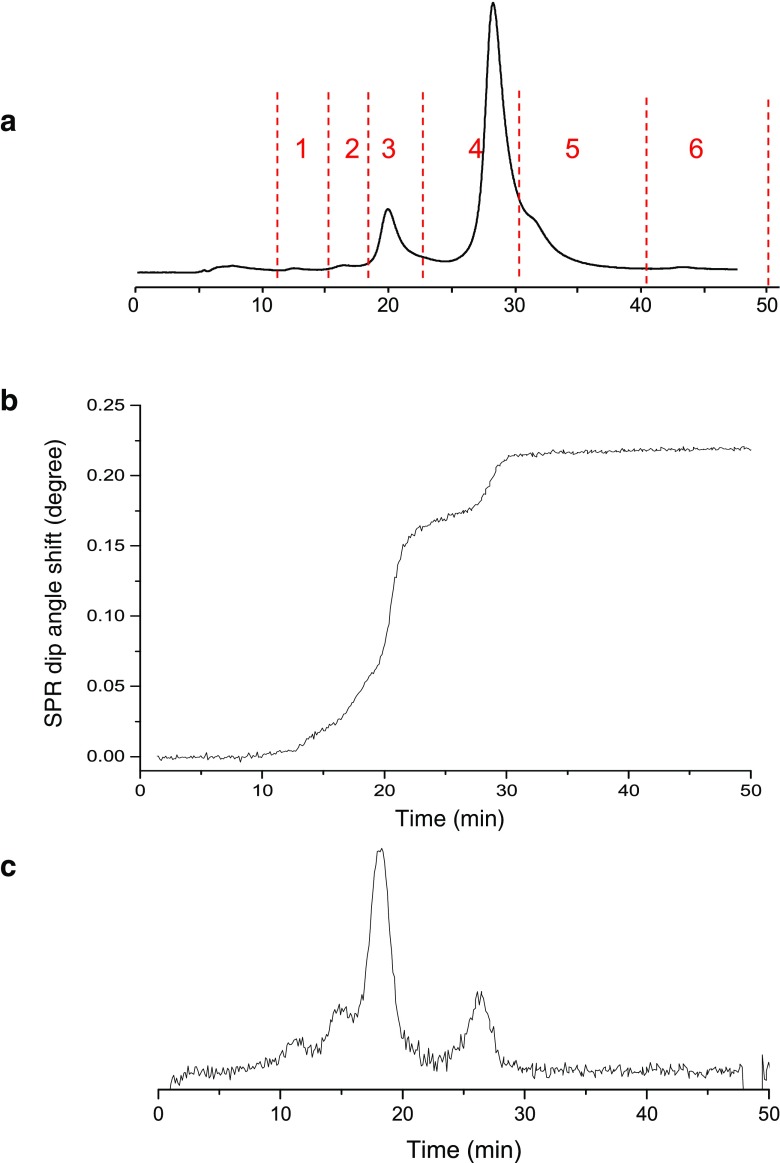
CEX-UV-SPR of trastuzumab. **a** UV chromatogram. **b** SPR sensorgram. **c** First derivative of SPR sensorgram

CEX is commonly used for the separation of antibody charge variants. In order to be able to evaluate the presence of charge variants in antibody preparations and their binding capacity towards HER2, CEX was directly coupled online to SPR. For post-column SPR affinity analysis, the sensor with immobilized HER2 was used. Before and after the main peak (retention time 28 min), acidic (peaks 1–3) and basic (peaks 5 and 6) variants were eluting, respectively. The acidic and basic variants are most probably corresponding to deamidated forms (lower pI) and non-clipped lysine variants, respectively (Fig. 5a) [33, 34]. The salt gradient causes a gradual refractive index change of the bulk effluent, resulting in a steady increase of the SPR response. In order to monitor the response caused by antibody-receptor binding, the signal from the reference channel (no receptor immobilized) was subtracted from the signal of the sample channel (ESM Fig. S4). Upon flushing with high concentrations of NaCl, HER2 on the surface of the sensor chip lost its activity (binding was no longer observed). Therefore, the CEX effluent was directed to waste after elution of the last peak (peak 6). The CEX-SPR sensorgram and its first derivative are depicted in Fig. 5b, c, showing the presence of several binding components in the sample. The first derivate shows that the charge variants represented by peaks 1, 2, and 3 are bound the surface of SPR and occupied most of the binding site on the surface. The fourth charge variant (peak 4) also shows binding to the surface of SPR; however, as a result of surface saturation, the binding of the other charge variants (peak 5 and 6) cannot be determined.

**Fig 6:**
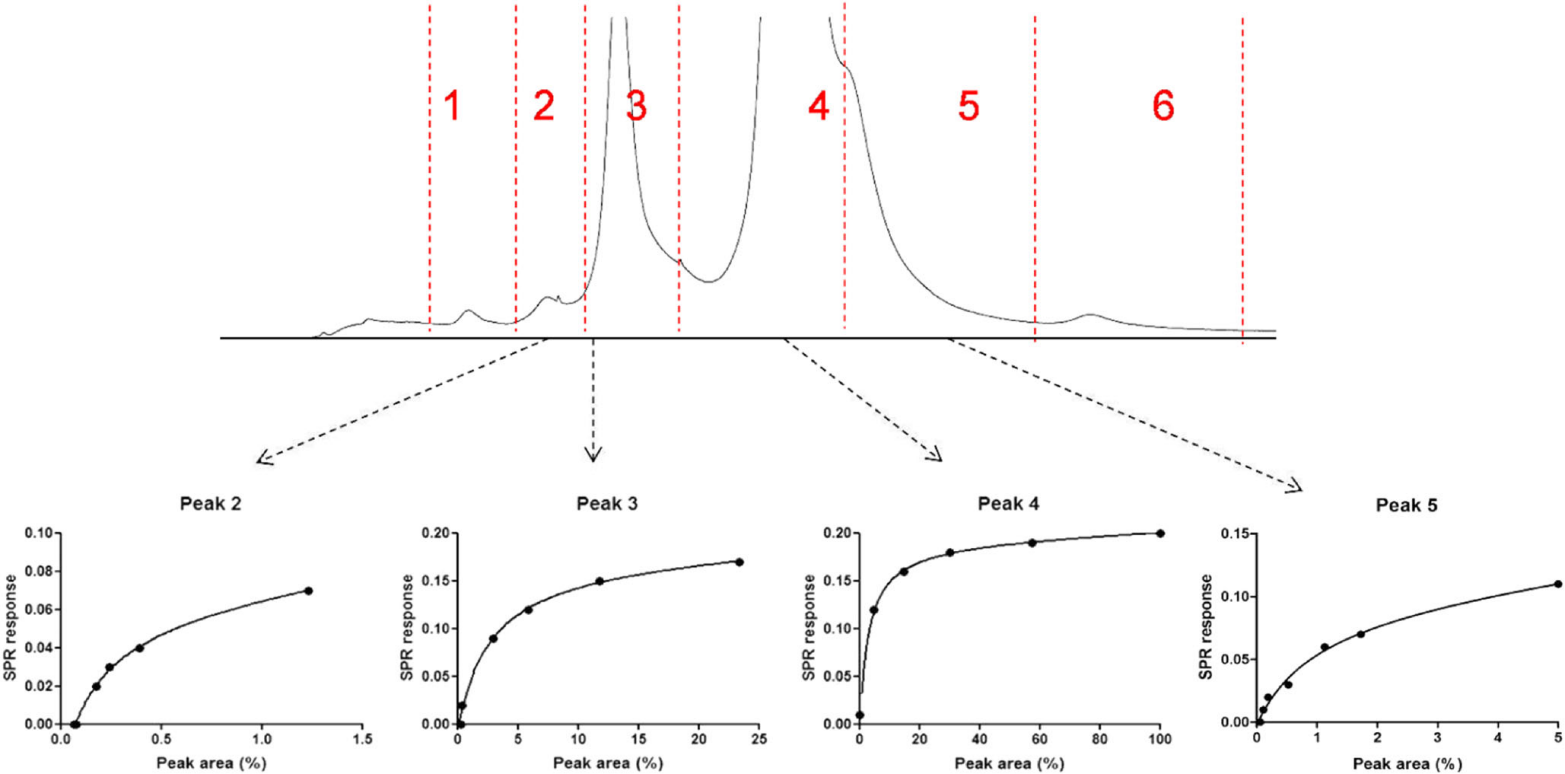
Heart-cutting CEX-SPR of trastuzumab (1–20 mg/mL injected in triplicate). Affinity curves of the CEX-separated heart-cutted peaks shown in the top UV chromatogram

In order to evaluate the ligand-binding capacity of separated charge variants of trastuzumab and to avoid saturation of the sensor surface by earlier eluted peaks, heart-cutting analysis was performed, directing the individual components to fresh sensor surface. Different concentrations of the antibody were injected, allowing construction of affinity curves (Fig. 6). The peak area per individual peak observed in the CEX-UV chromatograms was expressed relative to the peak area obtained for the main peak (peak 4) upon injection of the highest trastuzumab concentration (2 mg/mL). In order to compare the binding capacity of the charge variants, binding curves were plotted using the maximum SPR responses measured in the respective sensorgrams (*Y*-axis) versus the peak area percentage measured in the corresponding UV chromatograms relative to the main peak area (*X*-axis). Peaks 1 and 6 were too low in concentration to allow their binding capacity to be measured. At an injected trastuzumab concentration of 2 mg/mL, an elevated pattern in the affinity curve for the main peak (peak 4) was observed after about 25% of the peak was eluted with a maximum response value of 0.18 degree. Peak 3, at its maximum injected concentration (around 20% of the main peak), showed elevated pattern as peak 4, with an averaged maximum binding of 0.14 degrees. Comparison of peaks 5 and 3 showed similar behavior in their ligand-binding capacity (*R*_max_), with a maximum relative concentration of 5% compared to the main peak, showing an averaged maximum binding of 0.11 degrees. Peak 2’s binding pattern is also the same as that of other peaks with the maximum binding of 0.07 degrees at its highest binding concentration. The overlay of the affinity curves is shown in Fig. S5 (see ESM). These observations showed a similar binding pattern of the acidic and basic charge variants for HER2. The here-presented results are in line with a previous study of Dakshinamurthy et al. [34]. In order to be able to calculate kinetic parameters, the SPR signal should be corrected for actually probed concentration after LC elution; however, this is out of scope for this study. The results obtained with CEX-SPR shows the suitability of the proposed new methodology for monitoring of ligand-analyte binding capacities of the charge variants presents in pharmaceutical mixtures.

### SEC with parallel SPR and MS detection of trastuzumab and T-DM1

In order to achieve simultaneous structural assignment and affinity assessment of antibody samples, SEC was coupled in parallel to SPR and high-resolution MS using a post-column split. The mobile phase of sodium phosphate was replaced by 75 mM ammonium acetate (pH 6.9) in order to achieve compatibility with ESI-MS detection. Ammonium acetate at medium pH is sufficiently volatile and allows to preserve the higher-order structure of proteins, which is essential when interactions with immobilized receptors have to be probed by SPR analysis. The column effluent was split after the SEC column, so that a flow of 5 μL/min was directed to the ESI source and the rest to the UV detector followed by SPR detection (Fig. 1). This way, SPR and native MS analysis can be performed simultaneously, which offers the possibility to correlate the data obtained from both techniques. In addition, the low flow rate infused to the MS instrument favors efficient ionization of the analyzed antibodies.

**Fig 7:**
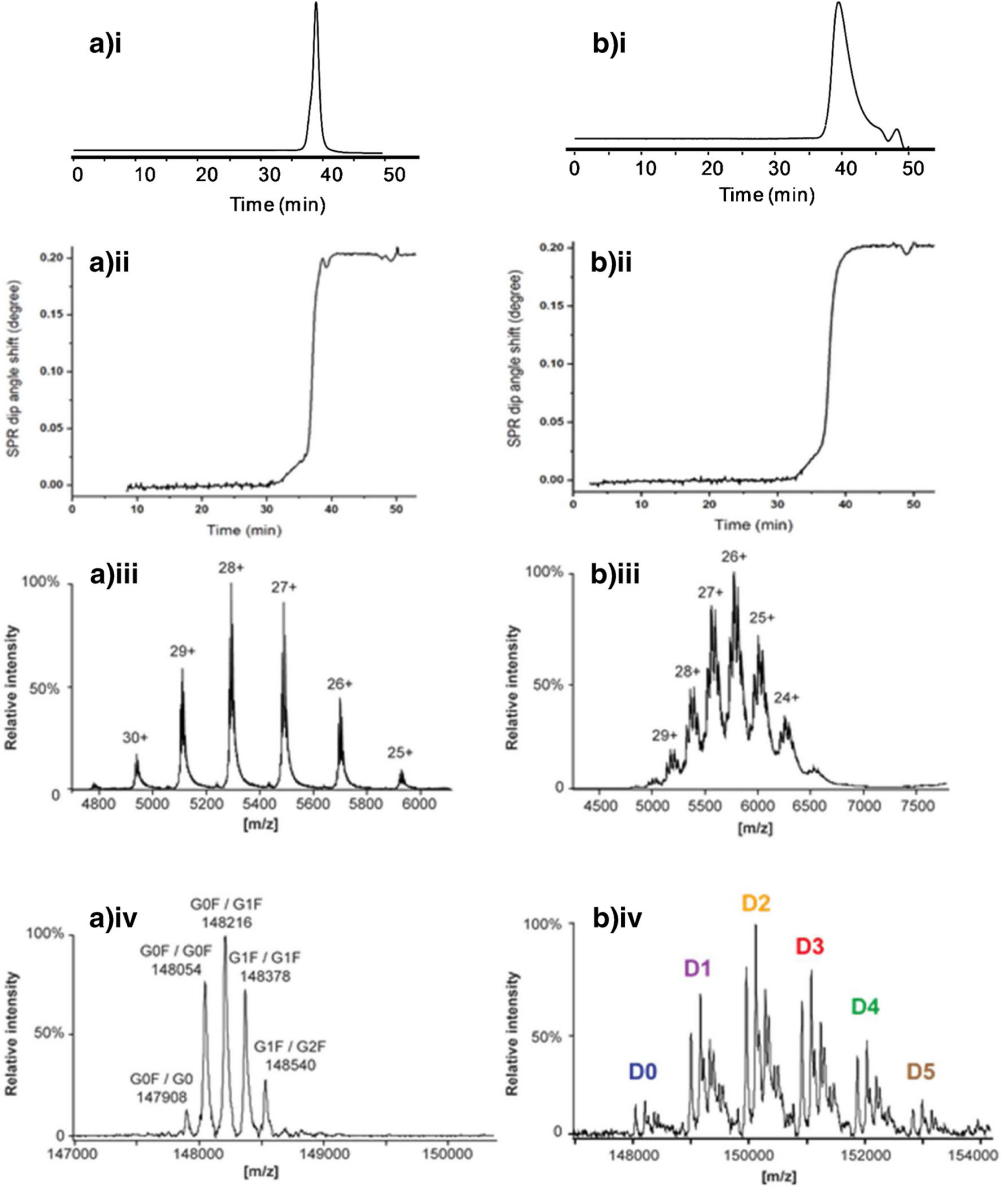
The SEC-SPR-MS analysis of (a) trastuzumab and (b) T-DM1 sample. A single run provides (i) UV chromatogram, (ii) SPR sensorgram, (iii) the raw, and (iv) deconvoluted mass spectrum

Results of SEC-UV-SPR/MS of trastuzumab and T-DM1 are depicted in Fig. 7. The obtained SEC-UV chromatograms (Fig. 7(a.i, b.i)) and the corresponding SPR sensorgrams (Fig. 7(a.ii, b.ii)) clearly demonstrate the interaction of the antibodies with the immobilized HER2 can be monitored by SPR efficiently when using ammonium acetate in the mobile phase. For the peak of trastuzumab, mass spectra comprised charge states ranging from 25+ to 30+ (Fig. 7(a.iii)). These charge states are in agreement with other studies, indicating that the trastuzumab is maintained in its native structure [35]. The deconvoluted mass spectrum revealed the presence of the five major glycoforms of trastuzumab (Fig. 7(a.iv.)), which has one *N*-glycosylation site on each heavy chain. Intact antibody analysis permits the assessment of the combination of glycans present on one antibody molecule. The theoretical and experimental mass comparison and the mass deviations over the different charge states, ranging from 0 to 1.3 Da depending on the glycoform, showed consistent performance of the high-resolution MS instrument and confident mass assignments (ESM Table S2). The satisfying mass accuracies can also attribute to the implementation of SEC which provides inline removal of sample salts that might lower MS resolution [36]. The mass spectrum allows estimation of the relative abundance of each glycoform which was in agreement with values commonly observed for trastuzumab formulations [37]. The overall results obtained indicate the potential ability of SEC-SPR/MS to correlate antibody/receptor interaction changes to antibody structural modifications, including glycosylation.

SEC-UV-SPR/MS was also performed for T-DM1, showing clear HER2 interaction. In addition, MS data provided detailed structural information on T-DM1. The charge states detected which ranged from 24+ up to 29+ are consistent with the preservation of the native structure of T-DM1 [38]. The MS data for T-DM1 exhibited an increased complexity (Fig. 7(b.iii)), allowing the assignment of antibody variants with different numbers of conjugated DM-1 drugs. Furthermore, the main glycoforms could be assigned for each T-DM1 conjugate (Fig. 7(b.iv)). The comparison of the theoretical mass and the experimental mass, in addition with the calculated mass deviations, proved the unambiguous identification of T-DM1 variants (Table 1).

**Table 1:** Comparison of the theoretical and experimental mass with calculated mass deviations for the analysis of T-DM1 using native SEC-SPR-MS.

Experimental mass (Da)	Number of conjugated drugs	Identified glycoform	Theoretical mass (Da)	Δm (Da)
148,052.50	0	G0F/G0F	148,054.06	1.56
148,215.60		G0F/G1F	148,216.20	0.60
149,005.00	1	G0F/G0F	149,012.59	7.59
149,170.00		G0F/G1F	149,174.73	4.73
149,335.00		G1F/G1F	149,336.87	1.87
149,820.70	2	G0F/G0	149,824.98	4.28
149,965.00		G0F/G0F	149,971.12	6.12
150,130.00		G0F/G1F	150,133.27	3.27
150,291.30		G1F/G1F	150,295.41	4.11
150,450.60		G1F/G2F	150,457.55	6.95
150,923.20	3	G0F/G0F	150,929.66	6.46
151,088.20		G0F/G1F	151,091.80	3.60
151,251.30		G1F/G1F	151,253.94	2.64
151,412.50		G1F/G2F	151,416.08	3.58
151,881.30	4	G0F/G0F	151,888.19	6.89
152,046.30		G0F/G1F	152,050.33	4.03
152,205.70		G1F/G1F	152,212.47	6.77
152,846.90	5	G0F/G0F	152,846.73	0.17
153,000.70		G0F/G1F	153,008.87	8.17
153,163.80		G1F/G1F	153,171.01	7.21

## Conclusions

An LC-SPR method is presented which allows separation of protein sample components (based on size or charge) prior to monitoring their affinity towards an immobilized antigen on the surface of the SPR sensor. The therapeutic antibody trastuzumab and the ADC T-DM1 were used as test samples for evaluation of developed methodology. Binding of the antibodies to the extracellular binding domain of HER2, which was immobilized on the gold sensor surface, was assessed. Firstly, SEC-SPR was explored for monitoring the affinity of components in native and stressed antibody samples. SEC allowed separation of the monomer antibody from its aggregates. Stressed T-DM1 formed three times more aggregates as compared to trastuzumab. Online SPR detection showed that aggregates and monomers have similar affinity towards HER2. Interestingly, the aggregates showed a different affinity curve (quasi sigmoidal pattern), which could be due to the presence of multiple binders with different binding affinities. Subsequently, a CEX-SPR method was developed for monitoring the effect of charge variants of trastuzumab on their binding capacities towards HER2. Several acidic and basic variants were separated, but exhibited no significant difference. In order to be able to evaluate the presence of charge-variant components of antibody samples and their binding capacity towards HER2, CEX was coupled to SPR. Overall, the obtained results demonstrate the feasibility of developed methodology for the maximum binding capacity monitoring of size and charged variants in pharmaceutical samples. Finally, parallel MS detection was added to the LC-SPR setup. The feasibility of the LC-MS/SPR system was demonstrated by analysis of the test samples, providing information on antibody glycoforms and/or determination of the drug-to-antibody ratio (DAR), while simultaneously monitoring binding of eluting species to HER2.

Overall, the hyphenated system represents a powerful analytical platform which in principle allows the correlation of affinity changes with structural modifications of therapeutic proteins, like mAbs and ADCs. The possibility to achieve, in a single experiment, such a structure-function characterization can be highly useful during research and development for instance. The versatility offered by the SPR instrument gives the opportunity to study the affinity of mAbs to different types of receptors. The here-presented method could be useful during biopharma lead optimization processes and for batch-to-batch comparisons of mAb and ADC products. To obtain correct binding kinetics and affinity parameters of separated sample components with LC-SPR, the data should be corrected for actual concentration considering multiple factors, such as mass transfer limitation, diffusion coefficients, and dynamic concentration profiles of the peaks.

## Electronic supplementary material

ESM 1 (PDF 204 kb)
