# Supplementary material for: Affinity profiling of monoclonal antibody and antibody-drug-conjugate preparations by coupled liquid chromatography-surface plasmon resonance biosensing
Source: Anal Bioanal Chem. 2018 Oct 17;410(30):7837–48. doi: 10.1007/s00216-018-1414-y (PMC6244757; doi:10.1007/s00216-018-1414-y)
Supplement: Supplementary file 1 — (PDF 204 kb) [file 216_2018_1414_MOESM1_ESM.pdf]

Analytical and Bioanalytical Chemistry

Electronic Supplementary Material

Affinity profiling of monoclonal antibody and antibody-drug-conjugate preparations by coupled liquid chromatography-surface plasmon resonance biosensing

Dina Lakayan, Rob Haselberg, Rabah Gahoual, Govert W. Somsen, Jeroen Kool

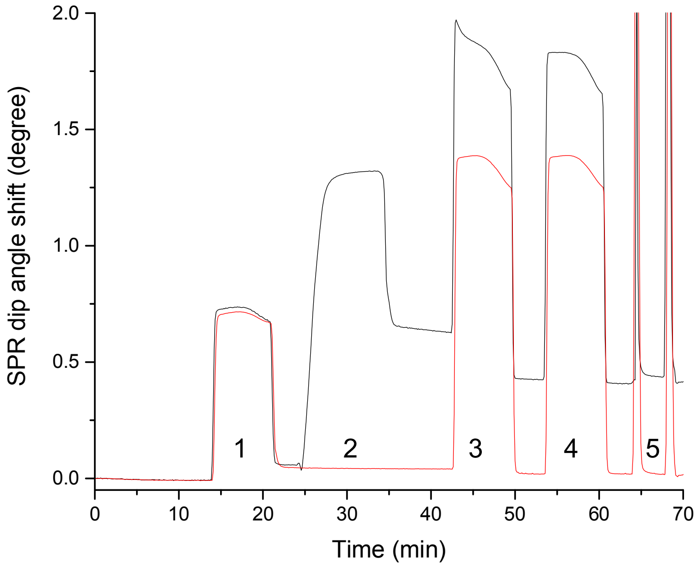

Fig. S1 Sensorgram of immobilization of the extracellular ligand binding domain of the HER2 receptor on carboxymethyl dextran sensor chip. 1. activation; 2. immobilization; 3. and 4. deactivation; 5. two regeneration steps

a.

[redacted]

b.

[redacted]

Fig. S2 Affinity curves obtained from standalone SPR analysis for a. Trastuzumab and b. TDM-1

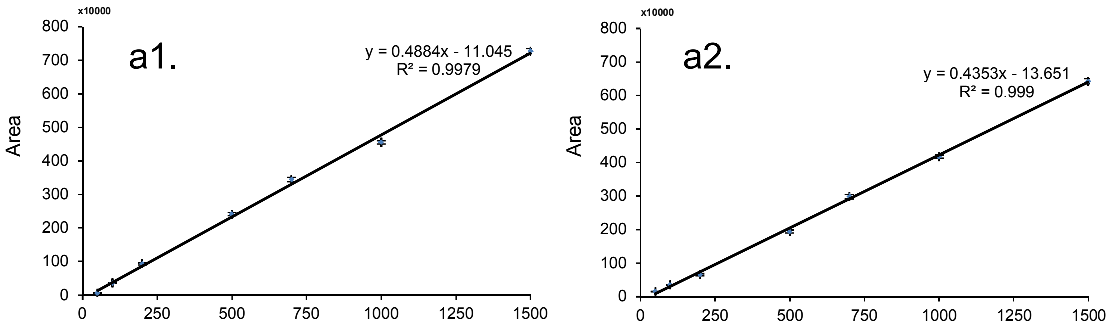

[redacted]

Fig. S3 SEC-UV-SPR of 1. trastuzumab and 2. TDM-1. a. calibration curves of injected antibody concentration vs. measured UV peak area; b. SPR affinity curves (SPR response versus peak concentration) for trastuzumab (1) and TDM-1 (2)

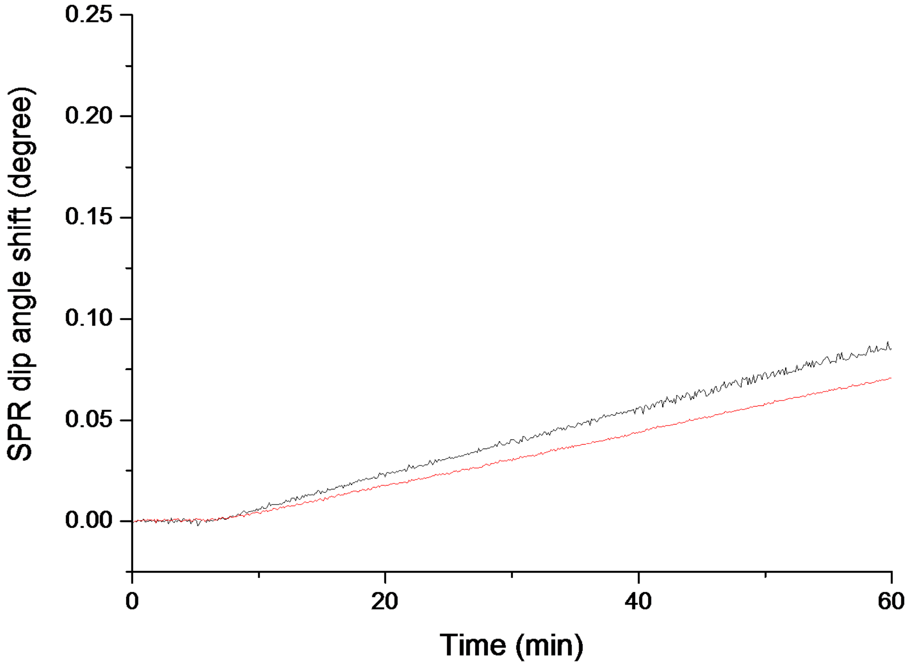

Fig. S4 SPR response monitored during CEX gradient. In order to correct for the gradient background signal, the reference channel signal (red line) was subtracted from the sample channel signal (black line)

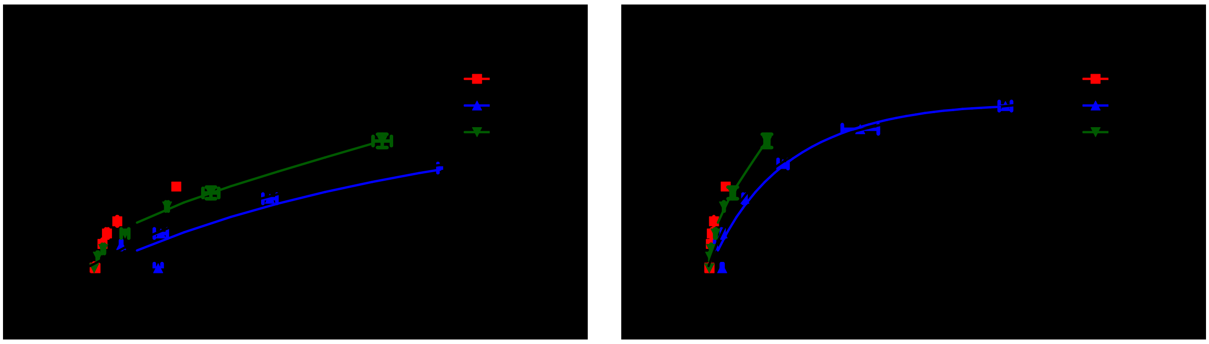

Fig. S5 SPR affinity curves of resolved charged variants of trastuzumab as obtained by CEX-SPR

Table S1 Kinetic parameters for the standalone SPR and SEC-SPR analysis of trastuzumab and TDM-1

Standalone SPR

Sample	ka (1/(M*s))	kd (1/s)	K_D1 (M)
Trastuzumab	(5.9 ± 1.3) × 10^4	(1.1 ± 0.2) × 10^{-4}	(1.8 ± 0.15) × 10^{-9}
TDM-1	(4.0 ± 0.7) × 10^4	(1.1 ± 0.1) × 10^{-4}	(2.7 ± 0.14) × 10^{-9}

SEC-SPR

Sample	ka (1/(M*s))	kd (1/s)	K_D1 (M)
Trastuzumab	(1.4 ± 0.8) × 10^3	(2.3 ± 0.4) × 10^{-5}	(1.6 ± 0.5) × 10^{-8}
TDM-1	(2.7 ± 1.2) × 10^3	(5.3 ± 0.7) × 10^{-5}	(1.9 ± 0.6) × 10^{-8}

Table S2 Comparison of the theoretical and experimental mass with calculated mass deviations for the analysis of trastuzumab using native SEC-SPR-MS

Experimental mass (Da)	Identified glycoform	Theoretical mass (Da)	Δm (Da)
147907.70	G0F / G0	147907.92	0.22
148053.80	G0F / G0F	148054.06	0.26
148216.20	G0F / G1F	148216.20	0.00
148378.60	G1F / G1F	148378.34	0.26
148539.20	G1F / G2F	148540.48	1.28
